# Relationship between Cardiometabolic Factors and the Response of Blood Pressure to a One-Year Primary Care Lifestyle Intervention in Metabolic Syndrome Patients

**DOI:** 10.3390/metabo12090861

**Published:** 2022-09-14

**Authors:** Elisa Marin-Couture, Marie-Josée Filion, Ryma Boukari, Khursheed Jeejeebhoy, Rupinder Dhaliwal, Paula Brauer, Dawna Royall, David M. Mutch, Doug Klein, Angelo Tremblay, Caroline Rhéaume

**Affiliations:** 1 Department of Kinesiology, Faculty of Medicine, Université Laval, Quebec City, QC G1V 0A6, Canada; 2 Centre de Recherche Nutrition, Santé et Société (NUTRISS), INAF, Quebec City, QC G1V 0A6, Canada; 3 Department of Family Medicine and Emergency Medicine, Faculty of Medicine, Université Laval, Quebec City, QC G1V 0A6, Canada; 4 Department of Nutritional Sciences and Physiology, University of Toronto, Toronto, ON M5S 1A4, Canada; 5 Canadian Nutrition Society, Ottawa, ON K1C 6A8, Canada; 6 Department of Family Relations & Applied Nutrition, University of Guelph, Guelph, ON N1G 2W1, Canada; 7 Department of Human & Health Nutritional Sciences, University of Guelph, Guelph, ON N1G 2W1, Canada; 8 Department of Family Medicine, University of Alberta, Edmonton, AB T6G 2T4, Canada; 9 Centre de Recherche de l’Institut Universitaire de Cardiologie et de Pneumologie de Québec (IUCPQ), Quebec City, QC G1V 4G5, Canada; 10 Centre de Recherche en Santé Durable—Vitam, CIUSS de la Capitale-Nationale, Quebec City, QC G1J 2G1, Canada

**Keywords:** blood pressure, cardiorespiratory fitness, waist circumference, lifestyle intervention, primary care, adverse responders, metabolic syndrome

## Abstract

Systemic hypertension has been recognized as a modifiable traditional cardiovascular risk factor and influenced by many factors such as eating habits, physical activity, diabetes, and obesity. The objective of this cross-sectional study was to identify factors that predict changes in blood pressure induced by a one-year lifestyle intervention in primary care settings involving a collaboration between family physicians, dietitians, and exercise specialists. Patients with metabolic syndrome diagnosis were recruited by family physicians participating in primary care lifestyle intervention among several family care clinics across Canada. Participants for whom all cardiometabolic data at the beginning (T0) and the end (T12) of the one-year intervention were available were included in the present analysis (*n* = 101). Patients visited the dietitian and the exercise specialist weekly for the first three months and monthly for the last nine months. Diet quality, exercise capacity, anthropometric indicators, and cardiometabolic variables were evaluated at T0 and at T12. The intervention induced a statistically significant decrease in waist circumference (WC), systolic (SBP) and diastolic (DBP) blood pressure, and plasma triglycerides, and an increase in cardiorespiratory fitness (estimated VO_2_max). Body weight (*p* < 0.001), body mass index (BMI) (*p* < 0.001), and fasting blood glucose (*p* = 0.006) reduction, and VO_2_max increase (*p* = 0.048) were all related to changes in SBP. WC was the only variable for which changes were significantly correlated with those in both SBP (*p* < 0.0001) and DBP (*p* = 0.0004). Variations in DBP were not associated with changes in other cardiometabolic variables to a statistically significant extent. Twelve participants were identified as adverse responders (AR) in both SBP and DBP and displayed less favorable changes in WC. The beneficial effects of the primary care lifestyle intervention on blood pressure were significantly associated with cardiometabolic variables, especially WC. These findings suggest that a structured lifestyle intervention in primary care can help improve cardiometabolic risk factors in patients with metabolic syndrome and that WC should be systematically measured to better stratify the patient’s hypertension risk.

## 1. Introduction

Systemic hypertension has been recognized as a modifiable traditional cardiovascular risk factor affecting almost 25% of the Canadian adult population [1]. High blood pressure (BP) is influenced by many factors such as eating behaviors, physical activity, diabetes, and obesity [1], especially visceral adipose tissue (VAT) [2,3,4], and cardiorespiratory fitness (CRF) levels [4,5,6]. Results from our laboratory previously demonstrated that individuals characterized with low VAT systematically displayed favorable values of systolic (SBP) and diastolic blood pressure (DBP), independent of their fitness level [7], and presented a reduced cardiometabolic risk [8]. VAT can be directly measured by using axial tomography scans, although waist circumference (WC) was shown to be a valid clinical marker of VAT that is easy to use in a clinical setting [9].

The current literature suggests that lifestyle interventions may reduce BP [10], body weight, and diabetes incidence [9,11,12,13,14], but seems to be less effective if not combined with pharmacotherapy in patients with dyslipidemia [15,16]. Nakao et al. [17] assessed the impact of a three-year lifestyle intervention, including a minimal exercise prescription, in patients with a metabolic syndrome (MetS) diagnosis. They showed significant reductions in WC, body mass index (BMI), SBP, DBP, and plasma triglyceride (TG) levels and an increase in HDL-cholesterol compared to control participants [17]. Gomez–Huelgas et al. [18] evaluated the effect of a three-year lifestyle intervention conducted by primary care providers in a randomized controlled trial among MetS patients and showed significant beneficial changes in WC, SBP, DBP, and HDL-cholesterol in the experimental group compared to control participants. To the best of our knowledge, few studies have investigated the relationship between BP and the response to lifestyle interventions and its association with other cardiometabolic factors in primary care settings following a lifestyle intervention in patients with MetS. Thus, the objective of the present study was to identify cardiometabolic factors that predict changes in BP induced by a one-year lifestyle intervention designed to reverse MetS. We hypothesized that an increase in maximal oxygen uptake (VO_2_max) and a decrease in WC would predict the changes of SBP and DBP in a pre-post structured intervention study such as the Canadian Health Advanced by Nutrition and Graded Exercise (CHANGE) program.

## 2. Materials and Methods

### 2.1. Study Population

**Figure 1:**
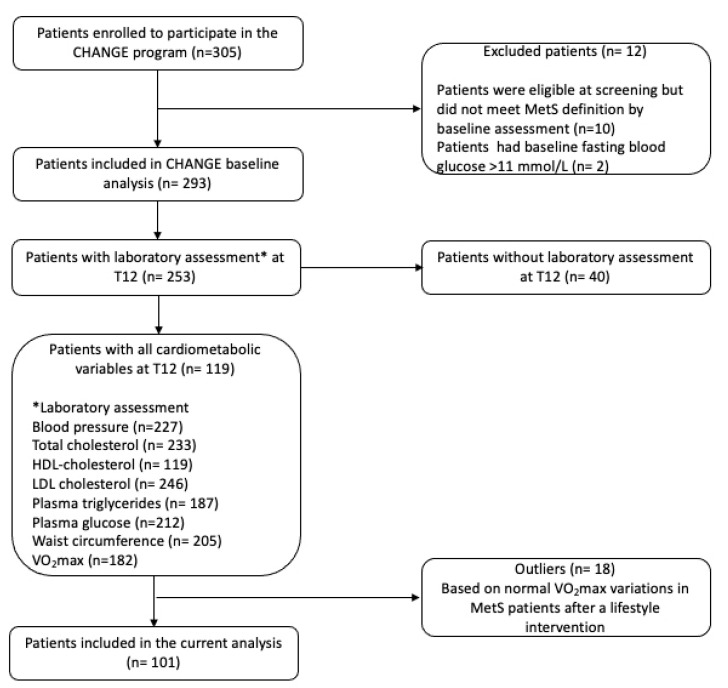
Flowchart of the patient’s eligibility in the present study. Abbreviations: CHANGE, Canadian Health Advanced by Nutrition and Graded Exercise; T12, end of intervention at 12 months.

The characteristics of participants in the CHANGE program have been previously reported [19]. The CHANGE program (website: https://www.metabolicsyndromecanada.ca/change-program, accessed on 12 July 2022) is a registered clinical trial (clinicaltrials.gov, ID: NCT01616563). Initially, 305 patients were recruited in the CHANGE program through their family physicians. There were 296 patients eligible to participate in the CHANGE program (eligibility criteria of the CHANGE program available in appendix 1 at www.cmajopen.ca/content/5/1/E229/suppl/DC1, accessed on 16 August 2022) from whom written and informed consent was obtained before inclusion [19]. At the end of the intervention, 256 patients had completed laboratory assessment and 119 of them had all cardiometabolic data available which are WC, total cholesterol, LDL-cholesterol, HDL-cholesterol, plasma triglycerides, fasting plasma glucose, SBP, and DBP [19]. There were also 182 patients for whom VO_2_max data was available [19,20]. Therefore, from the 119 patients study sample having all cardiometabolic data, 18 patients were considered as outliers based on their variations in VO_2_max between T0 and T12 values which did not agree with the normal increase [21,22] or decrease [23] variations in VO_2_max in MetS patients following lifestyle interventions according to the literature. In the present cross-sectional study, 101 patients were then analyzed according to the criteria of having all cardiometabolic data available at both T0 and T12 and showing normal VO_2_max variations between baseline and end of intervention values (Figure 1). As further described, the study sample was also subdivided into adverse responders (AR) (*n* = 10) and other participants (*n* = 91). Ethics approvals were obtained from Health Research Ethics Board-Biomedical (University of Alberta), Comité d’éthique de la recherche des Centres de santé et de services sociaux de la Vieille-Capitale, and the Institutional Review Board Services, A Chesapeake IRB Company (Aurora, Ont.) [19].

### 2.2. Experimental Design

**Figure 2:**
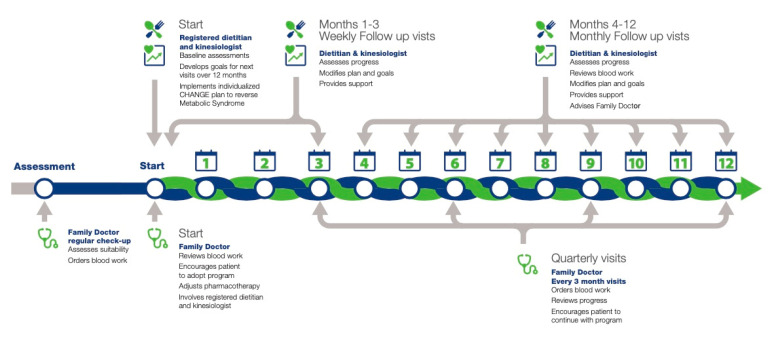
The CHANGE program protocol.

Participants were followed over a one-year lifestyle intervention involving a collaboration between family physicians, registered dietitians, and exercise specialists (Figure 2) [19]. Participants were given individualized diet and physical activity plans that were supervised by the registered dietitian and the exercise specialist [19,24] on a weekly basis for the first three months and monthly for the last nine months of the intervention [24]. As described elsewhere [24], the exercise specialist created an individualized fitness program based on the patient’s physical activity (PA) history, their PA preferences, their daily-life schedule, their health status, their stages of changes, and their real-life situation. An individualized diet plan based on the dietitian care map developed for dietary management of MetS patients [25] was used by the registered dietitian to promote weight loss (if feasible) at the beginning of the intervention followed by the management of diabetes or impaired glucose, hypertension and/ dyslipidemia with the use of a Mediterranean diet [26]. The family physician monitored the participant throughout the program and evaluated MetS variables at baseline (T0) and 12 months (T12) [19]. The involvement of the interdisciplinary healthcare practitioners in clinical settings allowed a close rigorous follow-up with the patients and, therefore, was safe for all participants in the CHANGE program. Diet quality, assessed by a Canadian version of the Healthy Eating Index (HEI-C) score, and exercise capacity were also measured at T0 and T12 [24,26].

### 2.3. Metabolic Measurements

BP measurement and blood sample collection were performed according to usual standardized procedures. Hemodynamic measures (resting BP and heart rate) were taken (Welch Allyn spot vital LXi, Hill-Rom Holdings Inc., Chicago, IL, USA). Blood samples were taken to measure blood lipid and glycemic profiles.

### 2.4. Anthropometric Measurements

Body weight was measured with a beam balance (Health o meter Professional scale, Biofix, McCook, IL, USA). Height was measured without wearing shoes while heels, buttocks, and the upper part of the back were in contact with a wall-mounted stadiometer. These measurements were used to calculate BMI. WC was measured according to standardized procedures at the midpoint between the last rib and the superior iliac crest.

### 2.5. Maximal Oxygen Uptake Assessment

The procedure of the Ebbeling single-stage walking test was used in the context of the CHANGE program, as previously reported [20]. Briefly, the test begins with a 4-min walking warm-up to achieve an estimated heart rate (HR) between 50% and 70% of the maximal HR estimated with the Karvonen equation [27]. Afterwards, the exercise specialist maintains the initially determined speed while increasing the treadmill grade to 5% for an additional 4-min period [27]. A steady-state HR (±5 bpm) needs to be achieved between the third and the fourth minute of the second period to complete the test, otherwise the test needs to be extended for an additional minute [27]. The VO_2_max is estimated by using a validated formula that considers age, sex, treadmill speed, and steady-state HR [27].

### 2.6. Statistical Analyses

Univariate analyses were conducted to show the anthropometric and metabolic characteristics of participants at both T0 and T12. A paired *t*-test was used to compare results obtained at T0 and T12 in all participants. Multiple regression analyses were performed to quantify the associations between changes in SBP and DBP and those of other measured variables using a multivariate regression model implemented in the REG procedure of SAS (SAS University Edition version 9.04.01M6P11072018). A Student’s *t*-test was used to compare baseline values and changes observed in AR for both SBP and DBP to those who favorably responded to the intervention. All values are expressed as mean ± SD and differences were considered significant at *p* < 0.05.

## 3. Results

The cardiometabolic variables of the 101 participants at T0 and T12 of the lifestyle intervention are presented in Table 1. A significant decrease in WC, blood TG levels, and both SBP and DBP was observed at T12. The intervention led to a significant increase in VO_2_max. This table also shows that favorable changes in body weight, BMI, as well as fasting plasma glucose and HDL-cholesterol, were achieved at the end of the study, although they did not reach statistical significance. 

The multivariate regression analyses considering the differences in cardiometabolic variables and the changes in SBP and DBP between T0 and T12 are presented in Table 2. Body weight (*p* < 0.0001), BMI (*p <* 0.0001), and fasting plasma glucose (*p* = 0.0061) reductions, as well as the VO_2_max increase (*p* = 0.0475), were related with the change in SBP (Table 2). Variations in DBP were associated with changes in body weight, BMI, and TG levels although not to a statistically significant extent (Table 2). WC was the only variable for which changes were significantly correlated with those in both SBP (*p* < 0.0001) and DBP (*p* = 0.0004) (Table 2).

There were 33 participants (32.7%) who increased their SBP in response to the CHANGE program, whereas an increase in DBP was observed in 30 subjects (29.7%). Furthermore, an increase in both SBP and DBP was observed following the intervention in 12 participants (11.9%) who were considered as AR in the present study. Table 3 shows the comparison between the cardiometabolic profile and the variations over time between AR and the other participants. At T0, SBP was significantly lower in AR and the same trend was found for DBP. As expected, the mean changes for both groups were significantly different for SBP (*p* < 0.0001) and DBP (*p* < 0.0001). The changes in WC between T0 and T12 were significantly lower in AR (−0.4 ± 3.5 cm) than in other participants (−4.7 ± 4.9 cm) (*p* = 0.0038).

**Figure 3:**
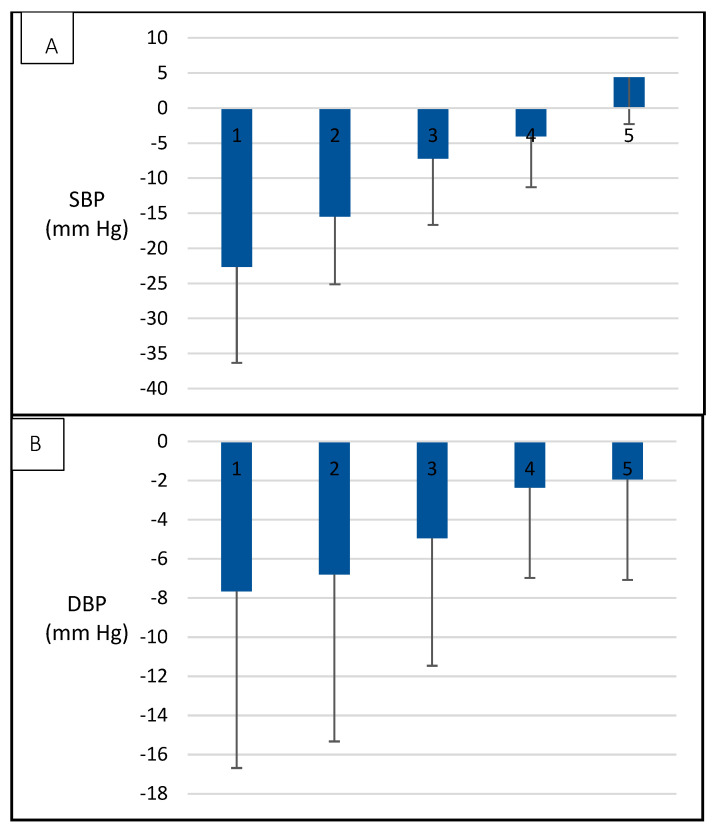
Association between changes in SBP and WC (Figure 2A) and DBP (Figure 2B) and changes in WC over a one-year lifestyle intervention. Groups 1 to 5 represent the distribution of participants according to the difference in WC between T0 and T12. 1, loss between 15.1 and 20 cm (*n* = 2); 2, loss between 15 and 10.1 cm (*n* = 9); 3, loss between 10 and 5.1 cm (*n* = 20); 4, loss between 5 and 0.1 cm (*n* = 46); and 5, gain between 0 and 4.9 cm (*n* = 18).

**Figure 4:**
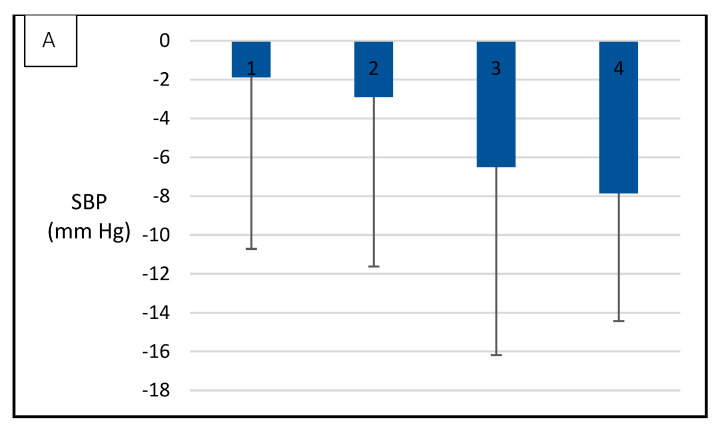

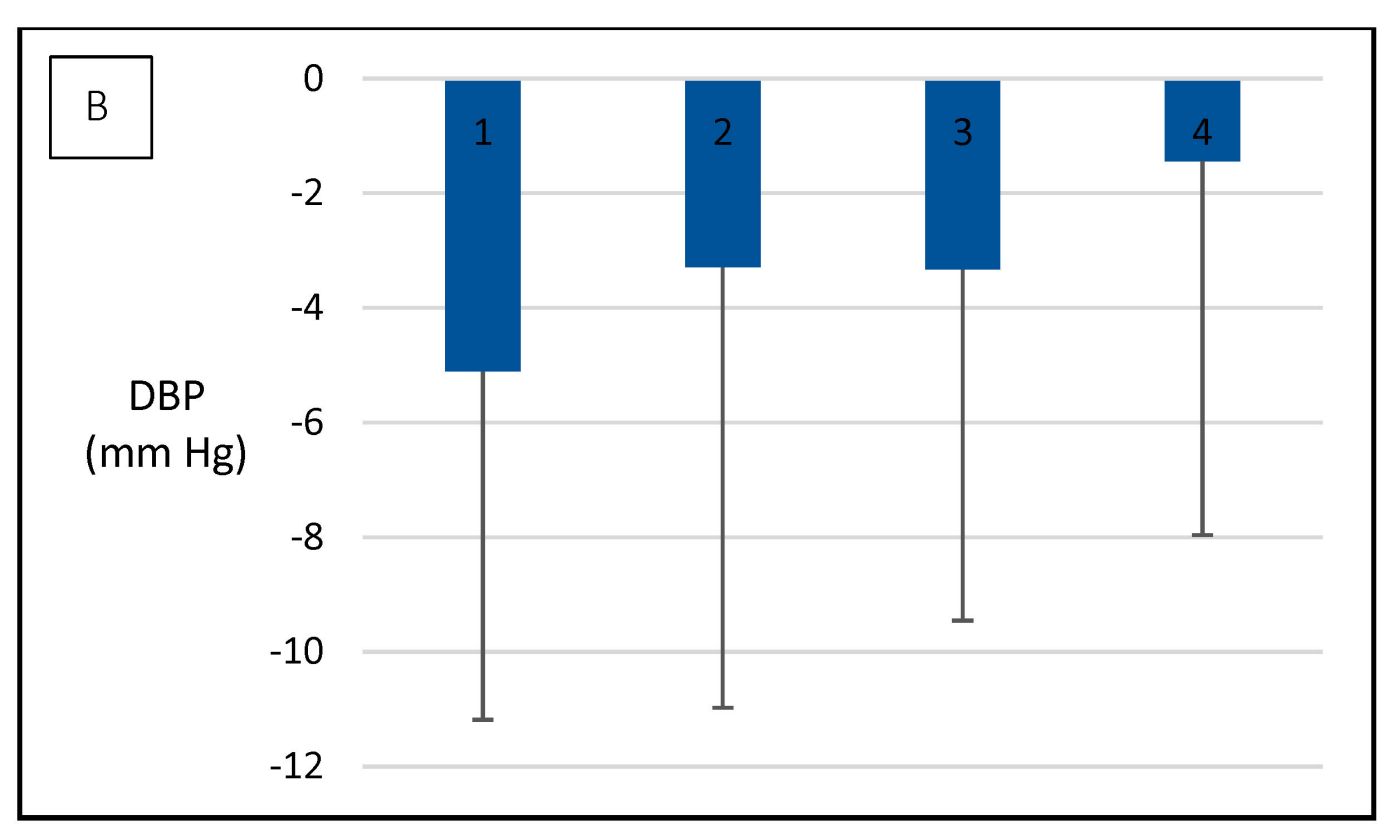
Association between changes in SBP (Figure 3A) and DBP (Figure 3B) and changes in VO_2_max over a one-year lifestyle intervention. Groups 1 to 4 represent the distribution of participants according to the difference in VO_2_max between T0 and T12. 1, loss between 0.1 and 3 mLO_2_/kg/min (*n* = 8); 2, gain between 0 and 2.9 mLO_2_/kg/min (*n* = 50); 3, gain between 3 and 5.9 mLO_2_/kg/min (*n* = 29); and 4, gain between 6 and 8.9 mLO_2_/kg/min.

Figure 3 presents mean values of SBP (Figure 3A) and DBP (Figure 3B) in participants classified into five groups according to WC changes. Participants who displayed less favorable changes in WC were also those who exhibited lower benefits in the response of SBP and DBP. Figure 4 shows an equivalent classification for changes in VO_2_max. Participants who achieved the greatest improvement in VO_2_max were also those who exhibited the most pronounced decrease in SBP. This association was not observed for DBP.

## 4. Discussion

Results of the present cross-sectional study suggest that the beneficial effects of a one-year lifestyle intervention on BP are associated with variations in cardiometabolic and anthropometric variables, especially those in WC in a real-life clinical and interdisciplinary context. Indeed, changes in WC were positively associated with those observed in both SBP and DBP. Changes in body weight, BMI, fasting plasma glucose, and VO_2_max were also positively associated with changes in SBP. In addition, a positive association was observed between body weight, BMI, and TG with changes in DBP, but not to a statistically significant extent. Moreover, the results showed that ~12% of the participants included in the present study were AR to the CHANGE intervention for both SBP and DBP. The AR in BP were also low or non-responders for changes in WC to the intervention. Thus, this cross-sectional investigation not only demonstrates the feasibility and the beneficial impact on cardiometabolic health of establishing individualized lifestyle intervention in primary care settings, but also reinforce the question regarding the use of precision medicine in a day-to-day medical practice. Indeed, the consideration of all patients in the present study showed that the interindividual response to lifestyle interventions might not generate the anticipated favorable adaptations to all. More studies regarding the impact of other avenues such as metabolomics are required to better understand the underlying effect of individualized interventions or treatments in patients.

The beneficial impact of a lifestyle intervention on the cardiometabolic profile has been documented [28,29,30], although only few studies have investigated this effect in primary care settings [11,12,13,14,31]. Höchsmann et al. [32] report that “there is a gap between obesity management and what is currently implemented in primary care”. This statement is also valid in the context of the management of systemic hypertension. The International Society of Hypertension and Hypertension Canada both recently revised their guidelines regarding systemic hypertension and mentioned that healthy lifestyle behaviors can prevent hypertension onset, reduce BP, and is considered as the first antihypertensive treatment [33,34]. Thus, implementation of structured lifestyle interventions in primary care settings by health professionals could be a good way to reach to the patients and prevent or treat systemic hypertension. Furthermore, supervised interventions such as the CHANGE program seem to induce beneficial lifestyle changes [19,35,36,37] that persist post-intervention [38].

WC reflects abdominal fat deposition and was shown to be a valid indicator of the accumulation of intra-abdominal adipose tissue which is composed of intra- and retroperitoneal fat [39]. Some evidence show that intraperitoneal fat better predicts insulin resistance and metabolic syndrome [40] and that patients displaying visceral fat, especially high intra-abdominal fat deposition, present higher risk of systemic hypertension [41,42] and deteriorated cardiometabolic profiles [43,44]. These results are in accordance with those reported in the present study and with some pathophysiological mechanisms that may explain the observed changes in BP and their relationship with those in WC, such as a sympathetic nervous system overactivation, a stimulation of the renin–angiotensin–aldosterone system, an alteration in adipose-derived cytokines such as leptin, and insulin resistance, as well as structural and functional renal changes [45,46]. In this regard, given the significant association between visceral obesity and the atheroinflammatory process, it is likely that targeting a reduction in WC with lifestyle interventions may provide improvements of the cardiometabolic profile such as favorable changes in BP and considerable reductions of cardiovascular disease risk.

Individuals displaying an adverse response in BP to the intervention were identified in the present study. Bouchard et al. [47] were probably the first investigators to report adverse response in common cardiometabolic variables following a well-controlled exercise program. They observed that one out of eight participants enrolled in the study presented an adverse response in SBP. Alvarez et al. [48] demonstrated that there were AR for WC, SBP, and DBP in 28 adult women participating in a 20-week exercise program. As indicated above, we also observed AR in BP to the program who had the particularity to display significantly lower values at baseline. Conversely, they tended to present greater BP values than other participants at the end of the study. Up until now, this unfavorable effect could not be explained, although it is worth emphasizing that these AR for BP tended to display less favorable changes in WC. More specifically related to the CHANGE program, the results from the investigations of Lowry et al. [49] and Lowry et al. [50] showed that individuals displaying certain genotypes or bioclinical measurements may benefit more from a lifestyle intervention than others and that, therefore, specific interventions should be considered for those predicted to be AR. Globally, our study further indicates that there exists a cluster of cardiorespiratory and metabolic variables predicting the outcome of a lifestyle intervention in MetS patient and that AR would benefit from healthcare professionals support to improve their cardiometabolic profiles.

The implementation of precision medicine in primary care settings has been widely discussed in the last few years for its potential to personalize patient care for individuals specific diseases or resistance to treatment [51]. The continued development of “-omic” technologies has shown great potential to uncover new therapies [51] while still considering an individual’s specific environment, socioeconomic status, medical history, or other conditions [52,53]. More specific to chronic disease management, metabolomics has now evolved in a way to show predictive, diagnostic, and prognostic abilities in the study of non-communicable diseases [54]. Because many chronic diseases are influence by complex interactions between lifestyle factors and genetics [54], the study of metabolomics promises to identify metabolic pathways that could be targeted for the prevention and/or treatment of non-communicable diseases. The current literature suggests that there is an existing association between PA and metabolites related to “branched amino acids, the TCA cycle, glycolysis, oxidative stress, insulin sensitivity, fatty acid mobilization, glucose, pyruvate, succinate and alanine, serine, glutamate, sarcosine, carnitines and kynurenine”, and that metabolomic responses to PA may differ between individuals [55]. Regarding dietary responses, some investigators have identified 15 salt-sensitive metabolites associated with a higher risk of systemic hypertension [56] and others have shown that adherence to a DASH-like diet reduced type 2 diabetes mellitus risk by modulating acylcarnitines and fatty acids [57]. Together, these findings suggest the additional value that may come from introducing metabolomics into day-to-day medical practice to better personalize a patient’s care. Moreover, systemic hypertension is a complex condition and is considered as a metabolic disorder for which the actual causes are still unclear but is largely influenced by an unhealthy lifestyle [58]. Because metabolomic profiles are capable of distinguishing hypertensive patients from individuals with a normal BP, more intervention studies to identity potential target metabolites need to be conducted in primary care settings to optimize the management of systemic hypertension [58]. In the present study, routine measurements helped to identify AR in BP that were also displaying less favorable changes in WC at the end of a one-year supervised lifestyle intervention. Even though the present study only measured common bioclinical markers, we hypothesize that examining the metabolome may reveal the basis for adverse responses to lifestyle interventions. Despite advances in knowledge in the field of lifestyle interventions, the use of metabolomics to help capture genetic information, lifestyle habits, and clinical outcomes remains limited and these investigations are necessary before it is adopted into routine medical practice.

A strength of this study is the demonstration of the feasibility in primary care settings of an individualized diet and fitness plan among MetS patients with the collaboration of family physicians, dieticians, and exercise specialists and reflects the real-life situation of health care partitioners promoting lifestyle interventions in their facilities to their patients. The examination of the profile of adverse BP responders supervised in a controlled clinical trial is another strength of this study. The study also has some limitations. Indeed, the absence of a control group not exposed to the lifestyle intervention limits to a certain extent the evaluation of the outcome of the CHANGE program. A randomized controlled trial would be necessary to further assess the causal relationship between the intervention and the variations in the cardiometabolic profile. The use of WC as a marker of VAT [7,8] may also be perceived as a limitation, although it has been shown to provide valid estimates of abdominal adiposity [9].

## 5. Conclusions

In summary, the beneficial effects of a structured lifestyle intervention (CHANGE program) on BP variations were significantly associated with changes in cardiometabolic variables, especially WC for both SBP and DBP in MetS patients in a primary care setting. Changes in CRF was also associated with those in SBP. Because the variations of WC seem to reflect the changes in SBP and DBP, we believe that health professionals in primary care facilities should systematically measure WC to stratify the patient’s systemic hypertension risk and is therefore strongly recommended. Additionally, our study documents for the first time the profile of AR in BP to a structured lifestyle intervention in MetS patients. Additional structured lifestyle studies are needed to characterize mechanisms that underlie variations in the response of BP and other cardiometabolic indicators to this type of intervention in the context of primary care settings and to target the specific metabolites to optimize the management of hypertension.

## Figures and Tables

**Table 1:** Anthropometric and metabolic characteristics of participants (*n* = 101).

Variable	Baseline (T0)	12 Months (T12)	% Change	*p* Value
Age	60.1 ± 9.3			
Height (m)	1.68 ± 0.1			
Body weight (kg)	88.0 ± 13.8	85.4 ± 13.7	−3.0	NS
Body mass index (kg/m^2^)	31.2 ± 3.4	30.3 ± 3.5	−2.9	0.0687
Waist circumference (cm)	105.9 ± 9.8	101.6 ± 10.5	−4.1	0.0040
Systolic blood pressure (mm Hg)	133.7 ± 13.0	129.1 ± 12.7	−3.4	0.0099
Diastolic blood pressure (mm Hg)	80.0 ± 13.0	76.8 ± 8.1	−4.0	0.0056
Total cholesterol (mmol/L)	4.83 ± 1.40	4.62 ± 1.28	−4.3	NS
HDL-cholesterol (mmol/L)	1.20 ± 0.27	1.24 ± 0.28	3.3	NS
LDL-cholesterol (mmol/L)	2.69 ± 1.13	2.58 ± 1.06	−4.1	NS
Plasma triglycerides (mmol/L)	2.07 ± 1.00	1.82 ± 0.71	−12.1	0.0380
Fasting plasma glucose (mmol/L)	6.32 ± 1.26	6.25 ± 1.19	−1.1	NS
VO_2_max (mLO_2_/kg/min)	33.1 ± 6.4	35.7 ± 6.44	7.9	0.0033

**Table 2:** Multivariate regression of the changes in cardiometabolic variables and changes in systolic and diastolic blood pressures (*n* = 101).

Variable	Systolic Blood Pressure	Diastolic Blood Pressure
Bodyweight	<0.0001	0.0760
Bodymassindex	<0.0001	0.0775
Waistcircumference	<0.0001	0.0004
Totalcholesterol	NS	NS
HDL-cholesterol	NS	NS
LDL-cholesterol	NS	NS
Plasmatriglycerides	NS	0.0984
Fastingplasmaglucose	0.0061	NS
VO_2_max	0.0475	NS

**Table 3:** Comparison of the cardiometabolic profile and variations over time between adverse responders (*n* = 12) in blood pressure and other participants (*n* = 89).

Variable	Baseline (T0)			End of Intervention (T12)			Variations between T0 and T12		
	AR	OP	*p* Value	AR	OP	*p* Value	AR	OP	*p* Value
	(*n* = 12)	(*n* = 89)		(*n* = 12)	(*n* = 89)		(*n* = 12)	(*n* = 89)	
Body weight (kg)	93.4 ± 16.7	87.4 ± 13.4	NS	92.2 ± 15.6	84.5 ± 13.3	0.0668	−1.1 ± 3.1	−2.9 ± 4.3	NS
Body mass index (kg/m^2^)	32.6 ± 4.4	31.1 ± 3.3	NS	32.2 ± 4.1	30.0 ± 3.4	0.0468	−0.4 ± 1.1	−1.0 ± 1.5	NS
Waist circumference (cm)	107.0 ± 11.0	105.7 ± 9.7	NS	106.7 ± 11.6	100.9 ± 10.2	0.0758	−0.4 ± 3.5	−4.7 ± 4.9	0.0038
Systolic blood pressure (mm Hg)	125.9 ± 14.4	135.0 ± 12.5	0.0222	133.0 ± 14.9	128.7 ± 12.3	NS	7.1 ± 3.9	−6.3 ± 9.7	<0.0001
Diastolic blood pressure (mm Hg)	75.9 ± 9.99	80.6 ± 8.7	0.0860	81.2 ± 8.5	76.2 ± 7.9	0.0467	5.3 ± 5.2	−4.4 ± 6.3	<0.0001
Total cholesterol (mmol/L)	4.55 ± 1.50	4.88 ± 1.38	NS	4.04 ± 1.6	4.71 ± 1.2	0.0891	−0.51 ± 0.89	−0.17 ± 0.63	NS
LDL-cholesterol (mmol/L)	2.26 ± 1.23	2.76 ± 1.11	NS	2.17 ± 1.19	2.65 ± 1.02	NS	−0.09 ± 0.55	−0.11 ± 0.56	NS
HDL-cholesterol (mmol/L)	1.21 ± 0.25	1.20 ± 0.28	NS	1.20 ± 0.24	1.24 ± 0.29	NS	−0.01 ± 0.13	0.04 ± 0.18	NS
Plasma triglycerides (mmol/L)	2.36 ± 1.46	2.10 ± 1.00	NS	1.92 ± 0.73	1.87 ± 0.89	NS	−0.45 ± 1.00	−0.22 ± 0.69	NS
Fasting plasma glucose (mmol/L)	6.34 ± 1.30	6.27 ± 1.39	NS	6.28 ± 1.14	6.19 ± 1.32	NS	−0.06 ± 0.47	−0.08 ± 0.96	NS
VO_2_max (mLO_2_/kg/min)	33.7 ± 4.5	33.1 ± 6.6	NS	36.8 ± 64.4	35.7 ± 6.7	NS	3.2 ± 2.6	2.6 ± 2.5	NS

Values are mean ± SD. Abbreviations: AR, adverse responders; OP, other participants.

## Data Availability

The datasets used and/or analyzed during the current study are available from the corresponding author on reasonable request.
